# Nonredundant, Highly Connected MicroRNAs Control Functionality in Breast Cancer Networks

**DOI:** 10.1155/2018/9585383

**Published:** 2018-05-29

**Authors:** Guillermo de Anda-Jáuregui, Jesús Espinal-Enríquez, Diana Drago-García, Enrique Hernández-Lemus

**Affiliations:** ^1^Computational Genomics Division, National Institute of Genomic Medicine, 14610 Mexico City, Mexico; ^2^Centro de Ciencias de la Complejidad, Universidad Nacional Autónoma de México, 04510 Mexico City, Mexico; ^3^Department of Biological Regulation, Weizmann Institute of Science, 7610001 Rehovot, Israel

## Abstract

Alterations to transcriptional regulation are an important factor in breast cancer. Noncoding RNA, such as microRNA (miR), have very influential roles in the transcriptional regulation of genes. Transcriptional regulation can be successfully modeled and analyzed using complex network theory. Particularly, interactions between two distinct classes of biological elements, such as miR and genes, can be approached through the bipartite network formalism. Based on bipartite network properties, it is possible to identify highly influential miRs in the network, such as those that have a large number of connections indicating regulation of a large set of genes. Some miRs in a network are nonredundant, which indicates that they are solely responsible of the regulation of a particular set of genes, which in turn may be associated to a particular biological process. We hypothesize that highly influential, nonredundant miRs, which we call *Commodore miRs* (Cdre-miRs), have an important role on the control of biological functions through transcriptional networks. In this work, we analyze the regulation of gene expression by miRs in healthy and cancerous breast tissue using bipartite miR-gene networks inferred from the Cancer Genome Atlas (TCGA) expression data. We observe differences in the degree, clustering coefficient and redundancy distributions for miRs and genes in the network, indicating differences in the way that these elements interact with each other. Furthermore, we identify a small set of five Cdre-miRs in the breast cancer network: miR-190b, miR-let7i, miR-292-b, miR-511, and miR-141. The neighborhood of genes controlled by each of these miRs is involved in particular biological functions such as dynein structure-associated processes, immune response, angiogenesis, cytokine activity, and cell motility. We propose that these Cdre-miRs are important control elements of biological functions deregulated in breast cancer.

## 1. Introduction

Breast cancer is a highly frequent cancer and one leading cause of death for women worldwide [1]. Aside from these important epidemiological aspects (and likely behind them) is the fact that breast cancer is a highly heterogeneous disease, both in its molecular origins and in its clinical manifestations; a fact that calls loudly for an improved understanding of the molecular mechanisms behind breast cancer development. Gene expression studies have provided with unprecedented information to characterize biomolecular activities leading to (or at least associated with) tumorigenesis. Recently, next generation sequencing has allowed us to accurately measure not only mRNA transcripts, but also regulatory noncoding RNA molecules such as microRNAs (miRs) and long noncoding RNAs (lncRNAs). miRs in particular have received a growing deal of attention due to their regulatory effects that seem to be fundamental for breast tumor establishment and progression [2].

In particular, the regulatory role of miRs seems to be central to processes involving cellular homoeostasis through processes such as apoptosis, proliferation, and migration that when deregulated give rise to well-known hallmarks of cancer [3–5]. Specific families of miRs playing either the role of “oncogenes” or “tumor suppressors” are commonly referred to as “oncomiRs” [6]. Specific miRNA regulation and coregulation patterns have been linked to oncogenic processes [7], specifically regarding breast cancer onset and evolution [2].

miR regulation at the transcriptional and posttranscriptional levels often occurs by inducing processes leading to mRNA destabilization [8]; transcriptome profiling is thus increasingly useful for the analysis of miR regulation in genome-wide settings. On the other hand, miRs have also been associated with other mechanisms of regulatory activity [9], in particular by widening target upregulation or downregulation [10]. These regulatory interactions are involved in mechanisms that may ensure biological robustness [11]. Regulatory relationships between miRs and genes (including transcription factors and other regulatory elements in the genome) are able to coexpress profiles that may be phenotype-inducing [12].

In order to improve our current knowledge on these matters, in particular regarding the transcriptional relation between miRNAs and their target genes (mRNAs), we propose the use of regulatory networks, using expression data from primary breast cancer tissue and matched control tissue. In our previous effort [13], we identified differences in the regulatory interactions of genes by other genes and by miRs; therefore, we decided to further explore these relationships using the bipartite graph formalism.

Bipartite networks are graphs composed by two disjoint sets of nodes and a set of edges such that each edge is surrounded by a node of each class. The two disjoint sets of nodes can be thought of as a *top* and *bottom* layers of nodes. The nature of bipartite graphs allows for the identification of topological parameters that describe both sets of nodes that are uniquely defined in this context. [14]. In this work, we will focus on three parameters: degree distributions, clustering coefficient, and redundancy coefficient.

### 1.1. Degree

The degree of a node is the number of edges that connect a node to its neighbors; it is thus the simplest measure of node connectivity. The degree distribution of a network is the most basic descriptor of a network [15]. In a bipartite network, the degree distribution is more informative if calculated for each set of nodes separately, as the connectivity of nodes in each set may be different. Thus, the degree distribution for a given set can be defined as follows:

(1) $$\begin{array}{c} P\left(K_{NodeSet}\right)=\frac{nk}{n}. \end{array}$$

### 1.2. Clustering Coefficient

The clustering coefficient of a node is a measure of the local density of connections in which a given node participates. The clustering coefficient of a network is thus a measure of the cohesiveness of the network [16]. The clustering coefficient in a bipartite node is defined as follows:

(2) $$\begin{array}{c} CC\left(u\right)=\frac{\sum_{\upsilon \in N\left(N\left(\upsilon \right)\right)}\left(\left|N\left(u\right)\cap N\left(\upsilon \right)\right|/\left|N\left(u\right)\cup N\left(\upsilon \right)\right|\right)}{\left|N\left(N\left(u\right)\right)\right|}. \end{array}$$

### 1.3. Redundancy Coefficient

**Figure 1:**
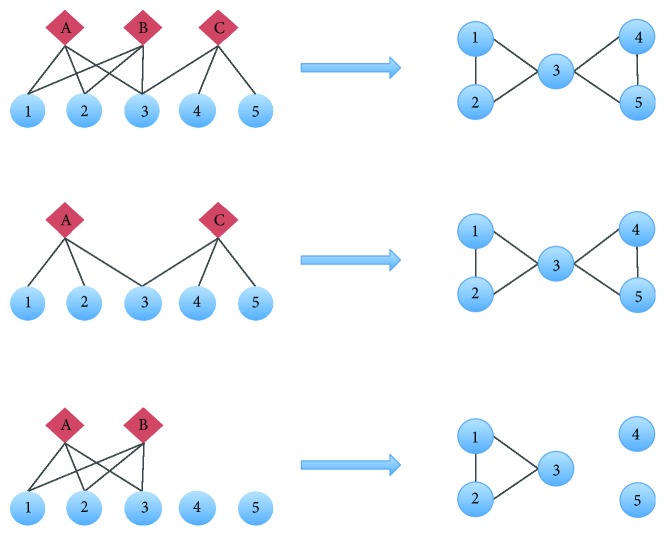
In this figure, we show a simple bipartite graph with 3 top nodes (A to C) and 5 bottom nodes (1 to 5). The projection of bottom nodes is shown on the right side. If node B is removed, no link in the projection is lost, as all bottom nodes connected through B are also connected through A; therefore, B is *redundant*. Meanwhile, removing node C causes the loss of connections of bottom nodes 4 and 5; therefore, C is *nonredundant*.

Nodes of the same type can only be connected by being connected through nodes of another type. The redundancy coefficient of a node is the fraction of pairs of neighbors of the said node that are both linked to other nodes [16]; it is a measure of the importance of a given node in a layer for the connection of the nodes in the other layer. Redundant nodes can be removed from their layer without causing a disconnection of the nodes in the other layer, as seen in Figure 1. The formal definition for the redundancy coefficient is as follows [17]:

(3) $$\begin{array}{c} RC\left(u\right)=\frac{\left|\left\{\left\{u,w\right\}\subseteq N\left(\upsilon \right),\exists \upsilon^{\prime }\neq \upsilon ,\left(\upsilon^{\prime },u\right)\in E,\left(\upsilon^{\prime },w\right)\in E\right\}\right|}{\left|N\left(\upsilon \right)\right|\left(\left|N\left(\upsilon \right)\right|-1\right)/2}. \end{array}$$

Noncoding RNAs, such as miRs, have a regulatory biological role. Meanwhile, genes may be involved in several biological activities, as they are translated into proteins which can have various functional roles. Usually, biological functions involve the interaction of several biomolecules to produce physiologically observable phenomena [18]. By regulating a set of genes, a given miR can in fact control a number of biological functions in a particular phenotype. We should take into account the following two considerations based on topological properties of a bipartite network:

- miRs with small neighborhoods (i.e., with low degree) are influencing few genes, being less likely to have effect on a biological function.
- miRs with high redundancy are less likely to be necessary for a given group of genes to be coregulated, as there are other miRs that will keep the group connected.

Therefore, we consider that highly connected, nonredundant miRs may have a major role in the control of biological functions through transcriptional networks: removal of these miRs would lead to the disconnection of a group of genes, which would involve a loss of the concerted regulation of this gene set, and in turn, of biological functionality. We have termed these miRs, *Commodore miRs* (Cdre-miRs).

## 2. Materials and Methods

### 2.1. miR-mRNA Bipartite Network Model

We constructed a bipartite network representing transcriptional regulation of genes by miRs in breast tissue, in the physiological and cancer states, hereafter referred to as the *healthy* and *cancer* groups.

We used gene and miR transcription profiles from samples from the Cancer Genome Atlas [19] breast cancer dataset [20]. Data processing and handling was performed following the pipeline previously implemented by our group [13] (Supplementary Material 1). The data processing pipeline included a differential expression analysis using DESeq2 [21] in order to identify the log fold change (LFC) of gene expression between the cancer and healthy groups.

Regulatory interactions between miRs and genes were inferred in terms of information theory. Mutual information (MI) [22] was calculated for each miR-gene pair, using the transcription profiles for either the healthy or cancer groups. Then, these interactions were filtered to conserve the 99.741%. MI calculation was performed using the engine of our in-house parallelized implementation of ARACNE [23].

Acknowledging that the canonical mode of action is that miRs regulate gene expression, we take this into account to interpret that mutual information interactions represent a directed relationship, from miR to gene; hence, higher values of mutual information indicate that the involved miR regulates directly the expression of the target gene.

### 2.2. Bipartite Network Analysis

Each network, healthy and cancer, was analyzed to identify general topological features, including number of miR and gene nodes, number of edges, and number of connected components. For each set of nodes, miR or genes, distributions were calculated for degree, clustering coefficient, and redundancy. Network analysis was performed using a combination of NetworkX for Python [16], igraph for R [24], and Cytoscape [25], as implemented previously [26].

### 2.3. Cdre-miR Identification

To identify highly influential, nonredundant Cdre-miRs, we separated all miRs in each network using two thresholds: a degree threshold of 100 and a redundancy threshold of 0.5.

### 2.4. Enrichment Analysis

For each Cdre-miR identified, we performed an over-representation analysis (ORA) of Gene Ontology (GO) biological processes [27, 28]. We performed the analysis using the WebGestalt [29] portal, with default parameters (number of genes in category between 5 and 2000, Benjamini-Hochberg multiple test adjustment, top 10 most significant processes).

## 3. Results

### 3.1. miR-Gene Bipartite Networks in Health and Cancer

**Figure 2:**
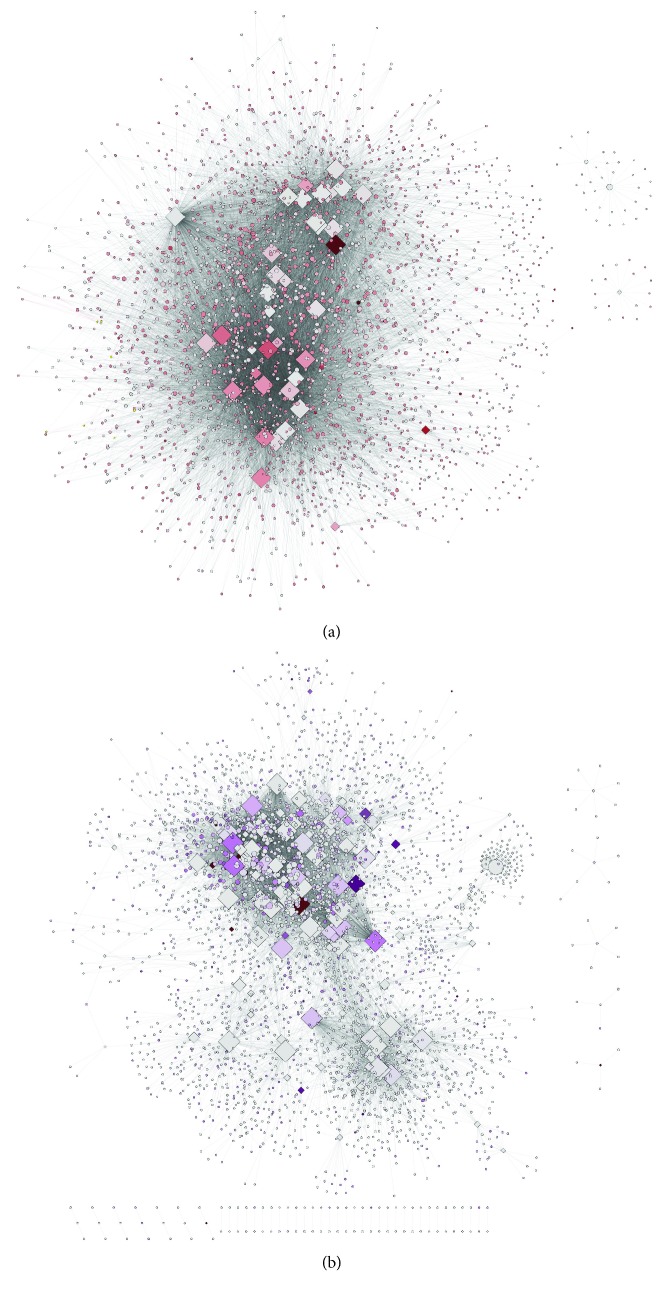
Network visualizations. (a) Healthy breast tissue. (b) Breast cancer tissue. Node color intensity in both networks is proportional to expression levels.

**Figure 3:**
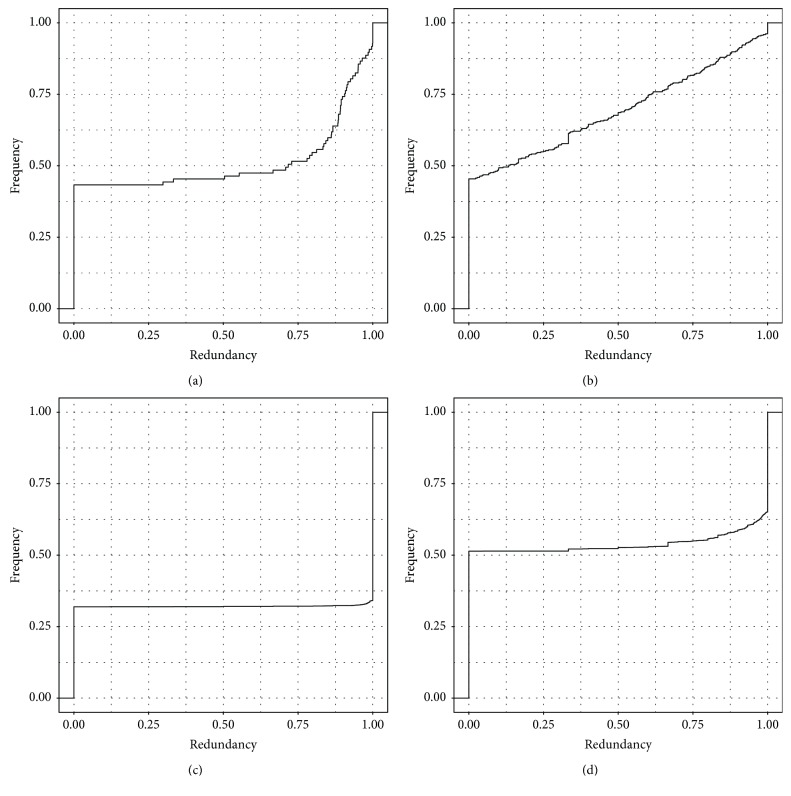
Redundancy coefficient cumulative frequency distribution plots. (a) miR, healthy. (b) miR, cancer. (c) gene, healthy. (d) gene, cancer. In each panel, *x*-axis represents redundancy coefficient values, and *y*-axis represents the normalized, accumulated frequency.

We constructed two comparable networks representing the two states: healthy and cancer.  Figure 2 shows a visualization of these networks.  Table 1 contains the basic descriptors for each network; these networks are also provided, as GML files, in Supplementary Material 2.  Figure 3 shows the cumulative frequency distribution for redundancy values for miRs and genes in healthy and cancer networks. Other distribution plots including degree and clustering coefficient of gene nodes are provided in Supplementary Material 2.

### 3.2. Identification of Cdre-miRs

**Figure 4:**
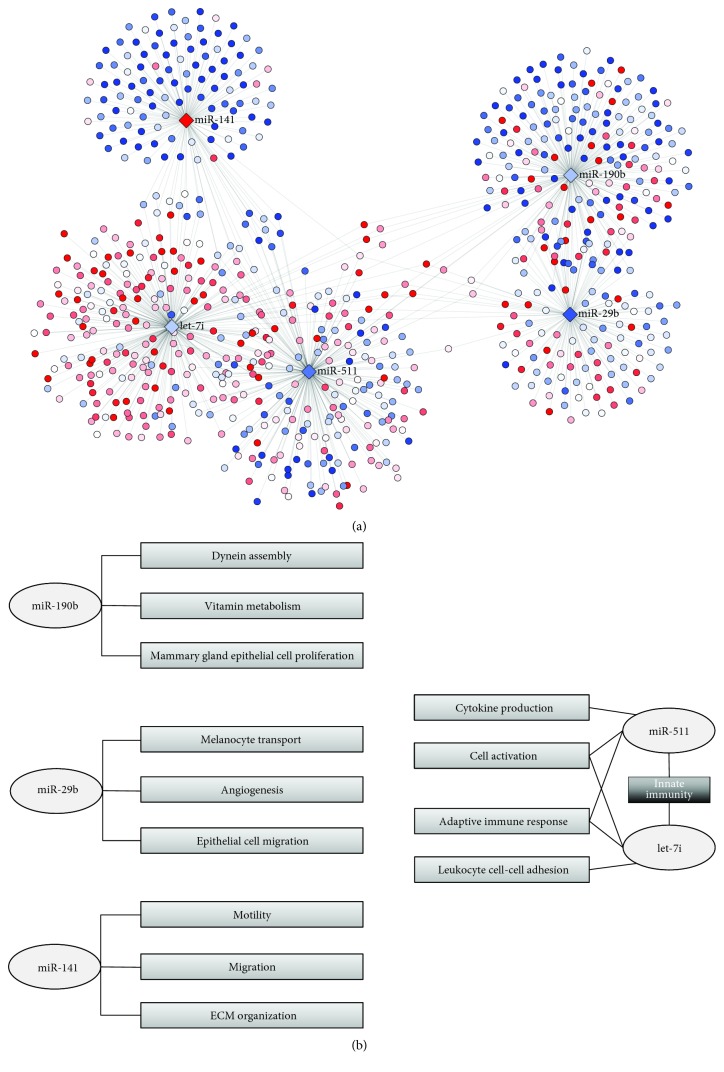
(a) shows the five Cdre-miRs and their gene neighbors. Nodes are colored in blue if subexpressed or red if overexpressed. (b) shows the GO biological processes enriched in each Cdre-miR neighborhood, in light grey. Additionally, the process enriched in the neighborhood intersection of mir-511 and let-7i, *innate immunity*, is shown in dark grey.

In Figure 4, we show a scatter plot of miRs, with the *y*-axis representing degree and the *x*-axis representing redundancy. We divide this plot in four sectors delimited by the thresholds selected to define high connectivity (degree larger than 100) and low redundancy (redundancy lower than 0.5). Five miRs were identified to be highly connected and nonredundant in the cancer setting: miR-190b, miR-let7i, miR-292-b, miR-511, and miR-141. Importantly, no Cdre-miRs were identified in healthy breast tissue.

Figure 4(a) shows the five Cdre-miRs and their first gene neighbors, colored by LFC. As it is expected, there is little overlap between the neighborhoods of each Cdre-miR; however, they are not completely isolated from each other, as there is a small subset of genes that are regulated by more than one Cdre-miR.  Figure 4(b) shows the 3 Gene Ontology biological processes more enriched in the gene neighborhoods of each Cdre-miR. Complete enrichment analysis results may be found in Supplementary Material 3. In this panel, we also include the enrichment of the neighborhood intersection of miR-511 and let-7i: the *innate immunity* process.

## 4. Discussion

Understanding the role of miR-gene regulation is crucial to unveil the mechanisms behind tumorigenesis and progression in breast cancer. Bipartite networks offer a powerful tool to analyze the behavior of these interactions and at the same time the structural and functional relevance of specific miRs in the transcriptional regulatory program.

**Figure 5:**
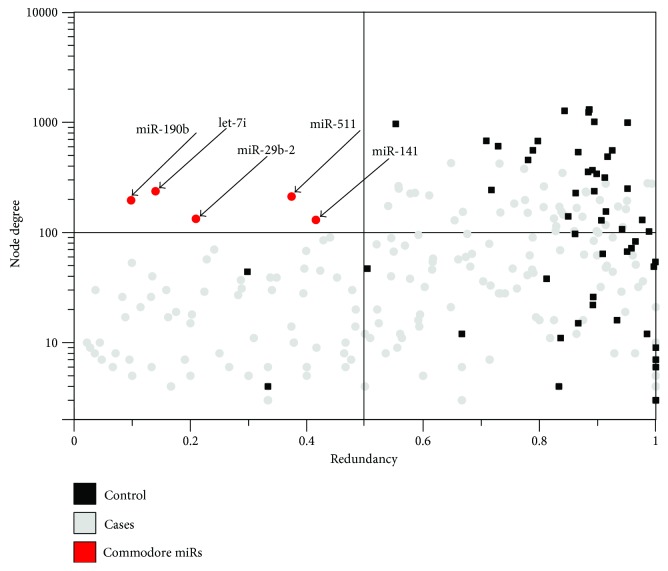
This scatterplot shows miR nodes in the healthy and cancer networks: grey dots for cancer and black squares for healthy. The *x*-axis represents the redundancy coefficient, and *y*-axis represents the degree of a miR. Only nodes with degrees larger than 2 are represented, as redundancy is not defined for nodes with a lower degree value. The plot is divided by lines representing thresholds for redundancy (0.5) and degree (100). The upper left sector of the plot is populated by five Cdre-miR nodes (red dots) with high connectivity and low redundancy.

In this work, we have shown that the bipartite networks representing miR-gene regulation in health and breast cancer exhibit notable structural differences. This can be observed in terms of structural parameters, including degree, clustering, and redundancy coefficients (as seen in Table 1, Figures 3 and 5, and Supplementary Material 4); the overall distribution of these parameters is qualitatively different in each network, which indicates that they have essentially different topologies. This result is in agreement with the widely understood notion that miR regulation is altered in cancer.

miRs are, on average, more connected in the health context than in cancer. They also happen to be more redundant, as seen in Figure 4. Biologically, this represents an abundance of joint miR regulation over sets of genes in health, which provides a mechanism that confers a structural robustness and a stronger maintenance of the transcriptional regulatory program in a normal tissue. Meanwhile, miRs in cancer tend to be less connected on average and also less redundant.

### 4.1. Cdre-miRs and Their Role in Breast Cancer

A major finding in this work is that the presence of highly connected, nonredundant miRs, which we define as *Commodores*, is a phenomenon only observed in the breast cancer network. Cdre-miRs emerge as important gene expression regulators, as each of them alone is responsible for the regulation of large groups of genes. Perhaps, more interesting is the fact that, through the regulation of these gene sets, each Cdre-miR is an important control element of specific biological processes.

For instance, miR-141 is a widely studied oncomiR [30–38]. Our group has previously observed that this miR is one of the most relevant mediators of structural processes in breast cancer and particularly involved in the epithelial-to-mesenchymal transition (EMT), as well as in the opposite mesenchymal-to-epithelial transition (MET) [13]. Here, we show that its functional importance is reflected by its crucial role in the miR-gene network. The processes that mir-141 controls through the regulation of its gene neighborhood, which includes genes such as VIM and ZEB1-2, are directly related to EMT-MET: motility, cell migration, and extracellular matrix (ECM) organization.

Another miR that has been previously studied in the context of breast cancer is miR-190b. Previously, it has been shown that this miR is associated with estrogen-positive breast cancer [39] and hormone therapy resistance [40]. However, its specific role in the pathogenesis of the disease has not been described. We identified mir-190b as a Cdre-miR that is associated to the regulation of dynein assembly, vitamin metabolism, and cell proliferation of mammary epithelial cells, a well-known hallmark of cancer. As a Cdre-miR, it is possible that deregulation of this miR is mechanistically involved in the acquisition of these distinctive cancer features.

Other important cancer features are controlled by miR-29b. This miR has been found in serum of patients with breast cancer [41] and overexpressed in cervical cancer [42]. In this work, we identify it as an important regulator of transport, angiogenesis, and epithelial cell migration, processes that are predominantly controlled by this miR.

### 4.2. Concurrent Regulation of Innate Immune Response by Cdre-miRs

So far, we have identified processes that are uniquely controlled by a single Cdre-miR. However, we also found processes that are controlled by two different Cdre-miRs, through the regulation of different gene neighborhoods. For instance, let-7i controls genes related to leukocyte cell-cell adhesion, adaptive immune response, and cell activation. Meanwhile, miR-511 regulates genes associated to cytokine production, cell activation, and adaptive immune response; that is, they each control related, but nonoverlapping, processes.

Importantly, the overlap between these two miRs is the largest observed between any pair of Cdre-miRs, but is less than 25% of the size of either miRs' neighborhood (53 out of 238 and 213, resp.). Perhaps more interestingly, by evaluating the enrichment of the intersection of these two Cdre-miRs, we identify that their jointly regulated genes highly overrepresent the innate immune response process, which is not significantly enriched by neither of neighborhood alone. This can be interpreted as an instance of a coregulated process that requires combined action of two different Cdre-miRs.

## 5. Conclusion

Using bipartite networks, we identified differences in the miR-gene regulation between healthy breast tissue and breast cancer. We christened those miRs that are highly connected and nonredundant as *Commodore miRs*. The emergence of Cdre-miRs is a network structural property that is only found in the context of breast cancer.

We identified five Cdre-miRs: miR-190, miR-29b, miR-141, miR-511, and let-7i that regulate specific biological processes. miR-190 is involved in cell structure, proliferation, and metabolism. miR-29b regulates cell transport, migration, and angiogenesis. miR-141 controls cell motility and extracellular matrix organization. miR-511 and let-7i independently regulate genes associated to cell adhesion and adaptive immune response through different gene sets. The latter shows that nonredundancy does not interfere with network robustness at a functional level.

Furthermore, innate immune response is a function which is not controlled by a single Cdre-miR, but it emerges in the context of the neighborhood overlap of miR-511 and let-7i. This is an example of how cooperation allows the emergence of new features that provide biological robustness to breast cancer. Identifying these elements is a step forward to identify actionable elements that provide controllability over the transcriptional networks.

Cdre-miRs may serve as novel biomarkers in breast cancer, which may be used to identify perturbation of biological functions in pathological phenotypes. Currently, therapeutic targeting of miRs in clinical oncology is limited; however, as technologies advance, the use of systems such as antagomiRs, microRNA mimics, and reporter systems will be available. In this context, we propose that Cdre-miRs may be attractive targets for the disruption of processes that are favorable for tumor growth and survival. With this in mind, Cdre-miRs may be an important element in the development of strategies for precision medicine in breast cancer. We strongly believe that further research in the role of Cdre-miRs in cancer shall provide a novel approach to understand the disease.

## Figures and Tables

**Table 1:** Bipartite network parameters.

Parameter	Healthy	Cancer
Nodes, miR	97	414
Nodes, gene	2967	3240
Edges	16,589	14,063
Average degree, miR	171.02	33.97
Average degree, gene	5.59	4.34
Average clustering coefficient, miR	0.28	0.18
Average clustering coefficient, gene	0.24	0.27

## Data Availability

The data used to support the findings of this study are available from the corresponding author upon request.

## Supplementary Materials

Supplementary Materials Supplementary Material 1. Compressed (Zip) file containing the graphic pipeline followed in this work. We use raw counts from RNA-seq and miRNA-seq for breast tumors and adjacent control (“healthy”) breast tissue from Genome Data Commons. MicroRNA preprocessing included mature count annotation, low-count filtering, and TMM normalization; RNA-seq preprocessing included mRNA annotation, RSEM normalization, and low-count filtering. This preprocessing is thoroughly described in [13]. These counts were integrated in an expression matrix which was used for a differential expression analysis (between cases and controls), and the inference of mutual information miR-gene networks. The final step is the identification of Cdre-miRs. Supplementary Material 2. Compressed (Zip) file containing GML representations of networks. Supplementary Material 3. Compressed (Zip) file containing ORA results for each Cdre-miR gene neighborhood. Supplementary Material 4. Compressed (Zip) file containing degree and clustering coefficient distribution plots.
