# Supplementary material for: Nonredundant, Highly Connected MicroRNAs Control Functionality in Breast Cancer Networks
Source: Int J Genomics. 2018 May 29;2018:9585383. doi: 10.1155/2018/9585383 (PMC5996465; doi:10.1155/2018/9585383)
Supplement: Supplementary Materials — Supplementary Material 1. Compressed (Zip) file containing the graphic pipeline followed in this work. We use raw counts from RNA-seq and miRNA-seq for breast tumors and adjacent control (“healthy”) breast tissue from Genome Data Commons. MicroRNA preprocessing included mature count annotation, low-count filtering, and TMM normalization; RNA-seq preprocessing included mRNA annotation, RSEM normalization, and low-count filtering. This preprocessing is thoroughly described in [13]. These counts were integrated in an expression matrix which was used for a differential expression analysis (between cases and controls), and the inference of mutual information miR-gene networks. The final step is the identification of Cdre-miRs. Supplementary Material 2. Compressed (Zip) file containing GML representations of networks. Supplementary Material 3. Compressed (Zip) file containing ORA results for each Cdre-miR gene neighborhood. Supplementary Material 4. Compressed (Zip) file containing degree and clustering coefficient distribution plots. [file 9585383.f1.zip › S_1.zip!/Pipeline_Cdre_2.pdf]

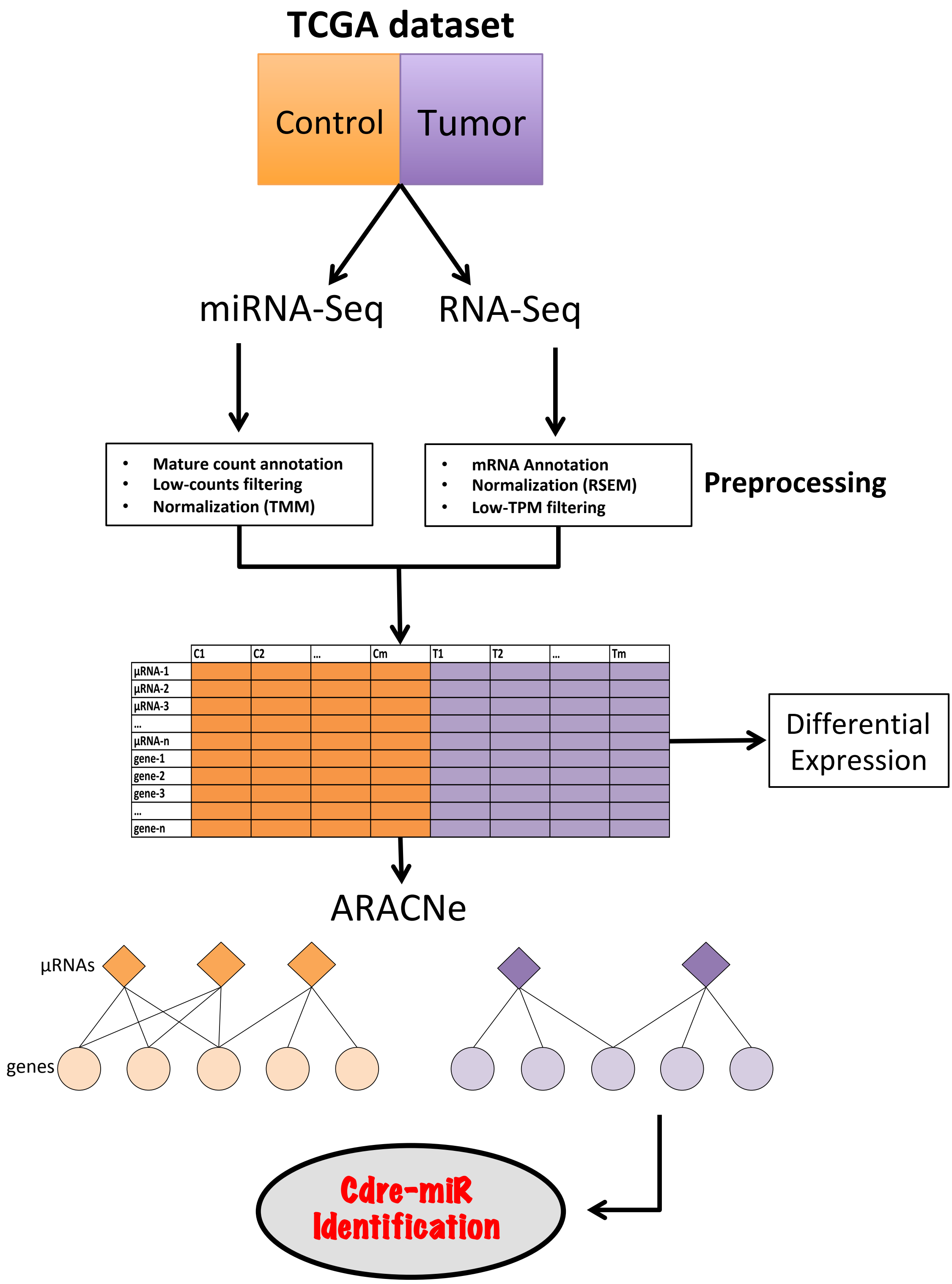

TCGA dataset
Control
Tumor
miRNA-Seq
RNA-Seq
Mature count annotation
Low-counts filtering
Normalization (TMM)
mRNA Annotation
Normalization (RSEM)
Low-TPM filtering
Preprocessing
C1
C2
...
Cm
T1
T2
...
Tm
µRNA-1
µRNA-2
µRNA-3
...
µRNA-n
gene-1
gene-2
gene-3
...
gene-n
Differential Expression
ARACNe
µRNAs
genes
Cdre-miR Identification
