# Supplementary material for: Nonredundant, Highly Connected MicroRNAs Control Functionality in Breast Cancer Networks
Source: Int J Genomics. 2018 May 29;2018:9585383. doi: 10.1155/2018/9585383 (PMC5996465; doi:10.1155/2018/9585383)
Supplement: Supplementary Materials — Supplementary Material 1. Compressed (Zip) file containing the graphic pipeline followed in this work. We use raw counts from RNA-seq and miRNA-seq for breast tumors and adjacent control (“healthy”) breast tissue from Genome Data Commons. MicroRNA preprocessing included mature count annotation, low-count filtering, and TMM normalization; RNA-seq preprocessing included mRNA annotation, RSEM normalization, and low-count filtering. This preprocessing is thoroughly described in [13]. These counts were integrated in an expression matrix which was used for a differential expression analysis (between cases and controls), and the inference of mutual information miR-gene networks. The final step is the identification of Cdre-miRs. Supplementary Material 2. Compressed (Zip) file containing GML representations of networks. Supplementary Material 3. Compressed (Zip) file containing ORA results for each Cdre-miR gene neighborhood. Supplementary Material 4. Compressed (Zip) file containing degree and clustering coefficient distribution plots. [file 9585383.f1.zip › S_4.zip!/cancer_ClusteringDot_gene.pdf]

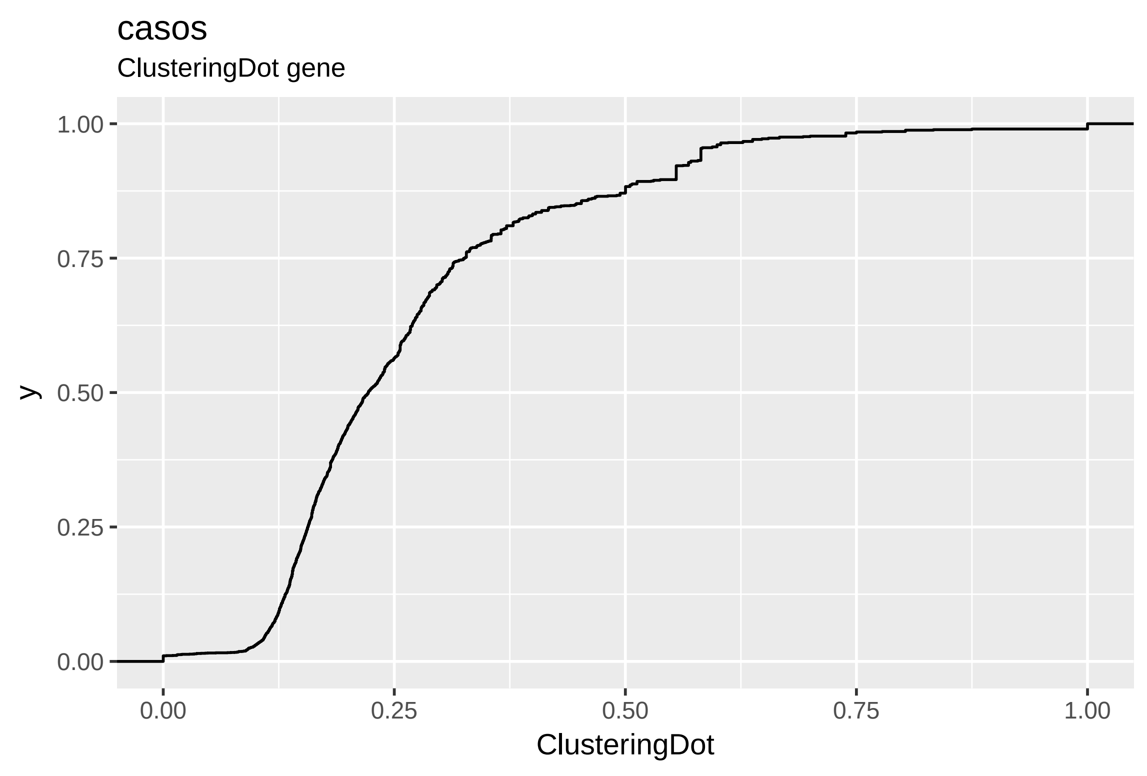
casos
ClusteringDot gene
1.00
0.75
0.50
0.25
0.00
y
0.00
0.25
0.50
0.75
1.00
ClusteringDot
